# Analysis of K-Ras Nuclear Expression in Fibroblasts and Mesangial Cells

**DOI:** 10.1371/journal.pone.0008703

**Published:** 2010-01-14

**Authors:** Isabel Fuentes-Calvo, Ana M. Blázquez-Medela, Eugenio Santos, José M. López-Novoa, Carlos Martínez-Salgado

**Affiliations:** 1 Unidad de Fisiopatología Renal y Cardiovascular, Instituto “Reina Sofía” de Investigación Nefrológica, Universidad de Salamanca, Salamanca, Spain; 2 Centro de Investigación del Cáncer-IBMCC, Universidad de Salamanca – Consejo Superior de Investigaciones Científicas, Salamanca, Spain; 3 Unidad de Investigación, Hospital Universitario de Salamanca, Salamanca, Spain; University of Illinois at Chicago, United States of America

## Abstract

**Background:**

Ras GTPases are considered cytoplasmic proteins that must be localized to cell membranes for activation, and there are few evidences of the presence of any Ras isoform in nuclei of eukaryotic cells.

**Methodology/Principal Findings:**

Using conventional antibodies and inmunocytochemistry, differential centrifugation and western blot, we have observed the putative presence of K-Ras isoform in the nuclei of fibroblasts and mesangial cells. In order to avoid cross-reactions with other Ras isoforms, and using antibodies against K-Ras (R-3400, H3845-M01, sc-30) or pan-Ras (05-516, OP40) in cells that only expressed the K-Ras isoform (fibroblasts obtained from H*-ras^−/−^*,N*-ras^−/−^* mice) we also detected some nuclear positive expression. To further probe the identity of nuclear K-Ras, we have generated K-Ras knockout (K*-ras^−/−^*) embrionary fibroblasts by mating of K*-ras^+/−^* heterozygote mice. Using specific antibodies, only H- and N-Ras isoforms were observed in the cytoplasm of K*-ras^−/−^* fibroblasts. However, both K-Ras4A and K-Ras4B positive signals were detected by immunocytochemistry and Western blot with two commercial antibodies (sc-522 and sc-521 against each isoforms, respectively) in both cytoplasm and nuclei from K*-ras^−/−^* fibroblasts.

**Conclusions/Significance:**

We show that the presence of K-Ras4B in fibroblast nuclei, already described by other authors, is probably due to a cross-reaction of the antibody with an undetermined nucleolar protein. Although this study also shows the possible nuclear expression of K-Ras isoform in fibroblasts or in mesangial cells, it also reveals the importance of being cautious in these studies about distribution of protein isoforms due to some important limitations imposed by the unspecificity of the antibodies or contaminations in cellular preparations.

## Introduction

Ras proteins control cell growth, proliferation and other aspects of cellular biology including senescence/cell cycle arrest, differentiation and survival, due to their ability to modulate transcription [1]. The classical Ras proteins (p21 Ras: H-, K- and N-Ras), together with M-Ras, R-Ras, Rap and Ral, are the prototype members of the Ras subfamily that is included in the Ras superfamily of small monomeric GTP-binding (G) proteins; this superfamily also includes the Rho, Ran, Rab, Rac, Rheb, Arf and Kir/ReM/Ras subfamilies [2]. p21 Ras proteins include three closely related members with a molecular mass of ∼21 KDa: H-Ras (or Ha-Ras), K-Ras (or Ki-Ras) and N-Ras. K-Ras occurs in two alternatively spliced forms: Ki(A)-Ras (or K-Ras4A) and Ki(B)-Ras (or K-Ras4B), deriving from *Kras-2* gene expression [2]. In mammals, these three functional *ras* genes are ubiquitously expressed in all organs and located in different chromosomes [3]–[5] but expression levels may vary between different cell types.

Several lines of evidence suggest the existence of unique roles for the three mammalian *ras* genes; gene targeting experiments have demonstrated that neither H-Ras nor N-Ras function are essential in the mouse: N-ras homozygous mutant mice grow normally [6]. In addition, disruption of H-Ras and N-Ras, individually or in combination, reveals dispensability of both loci for mouse growth and development [7]. In contrast, embryos homozygous for a mutation in K-*ras* die between 12 and 14 days of gestation, with foetal liver defects and evidence of anaemia [8]. Thus, K-*ras* is the only member of the *ras* gene family essential for mouse embryogenesis [8], [9].

Signal transduction down the Ras pathway has been generally considered to initiate at the plasma membrane. It is now clear that the plasma membrane does not represent the only platform for Ras signalling: genetically encoded fluorescent probes have revealed signalling on a variety of intracellular membranes, included the Golgi apparatus [10]. Thus, the members of the Ras family of proteins are considered cytoplasmic proteins that must be localized to the plasma or other intracellular membranes for activation, but there are only two studies showing the nuclear presence of Ras isoforms [11], [12]. Birchenall-Roberts et al. [12] has been the first and only group to describe the presence of K-Ras4B isoform in fibroblast nuclei.

In order to study the cellular distribution of the K-Ras isoform and its possible function, we have generated K-Ras knock-out (K-*ras* ^−/−^) embrionary fibroblasts by culturing K-*ras* ^−/−^ embryos obtained from mating of K-Ras heterocygous mice (K-*ras* ^+/−^). We have also analyzed K-Ras cellular distribution in H-*ras* ^−/−^/N-*ras* ^−/−^ fibroblasts, which only express the K-Ras isoform. In this study we show the putative presence of K-Ras isoforms in nuclei of human mesangial cells and fibroblasts from H-*ras* ^−/−^/N-*ras* ^−/−^ mice, whereas H- and N-Ras isoforms were absent. We hypothesize that the detection of the K-Ras4B isoform in nuclei of human fibroblasts previously described by Birchenall-Roberts et al. [12] is probably due to cross-reaction of the antibody used against K-Ras4B since we observed cytoplasmic and nuclear staining in K-*ras* ^−/−^ embrionary fibroblasts using the same antibody. This experimental work puts forward the need to be careful as well as provides useful information about antibodies specificity in studies about Ras isoforms.

## Results and Discussion

**Figure 1:**
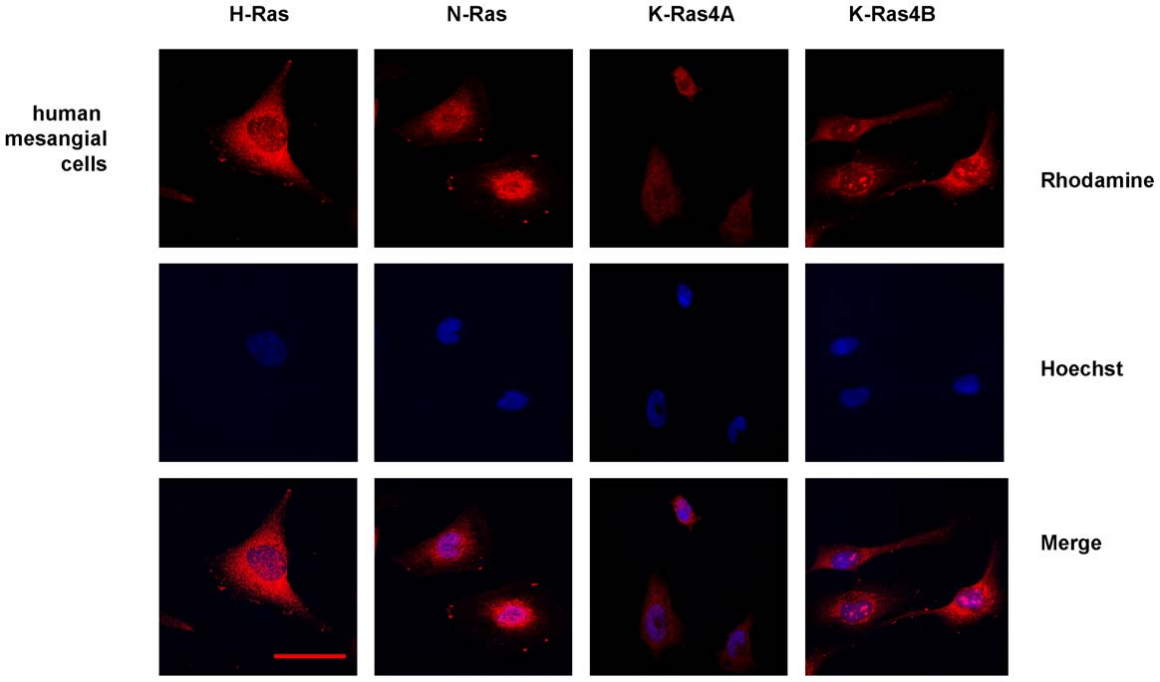
Immunofluorescence analysis of cellular distribution of Ras isoforms in human mesangial cells. Pictures show H-Ras, N-Ras, K-Ras4A and K-Ras4B expression in human mesangial cells. The rhodamine red-labeled proteins are Ras isoforms, while the blue-labeled staining is Hoechst-stained DNA. Magnification: 630×. Scale bar: 30 µm. Antibodies: H-Ras, sc-520; K-Ras4A, sc-522; K-Ras4B, sc-521; N-Ras, sc-519.

Cellular distribution of Ras isoforms was examined by immunofluorescence analysis in a confocal microscope. H-, N- and K-Ras isoforms are expressed in the plasma and intracellular membranes of human mesangial cells, but nuclei from these cells also showed positive staining for K-Ras4B isoform appearing as dense aggregates using the sc-521 antibody, which is supposedly specific for K-Ras4B isoforms (Figure 1). K-Ras4A staining detected with sc-522 antibody was fainter than K-Ras4B expression, accordingly with the fact that K-Ras4B is the predominant splice variant [13].

**Figure 2:**
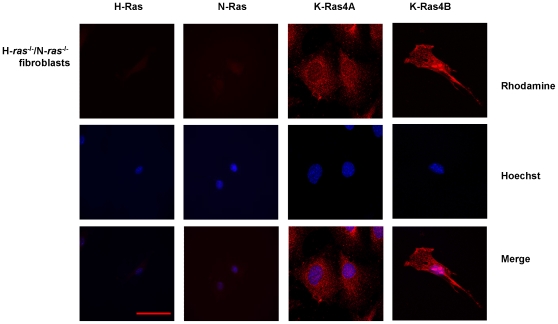
Immunofluorescence analysis of cellular distribution of Ras isoforms in H-*ras* ^−/−^/N-*ras* ^−/−^ fibroblasts. Pictures show H-Ras, N-Ras, K-Ras4A and K-Ras4B expression in fibroblasts. The rhodamine red-labeled proteins are Ras isoforms, while the blue-labeled staining is Hoechst-stained DNA. Magnification: 630×. Scale bar: 30 µm. Antibodies: H-Ras, sc-520; K-Ras4A, sc-522; K-Ras4B, sc-521; N-Ras, sc-519.

To exclude the possibility of cross-reaction between anti-K-Ras and other Ras isoforms, we assessed the expression of Ras isoforms in fibroblasts from H-*ras* ^−/−^/N-*ras* ^−/−^ mice using the same antibodies. Our results confirmed the lack of H- and N-Ras isoforms, the presence of K-Ras4A distributed along the plasma membrane and the expression of K-Ras4B in plasma membrane and nuclei; these results confirm that the pattern of expression observed in mesangial and fibroblast nuclei supposedly corresponded to K-Ras (Figure 2).

**Figure 3:**
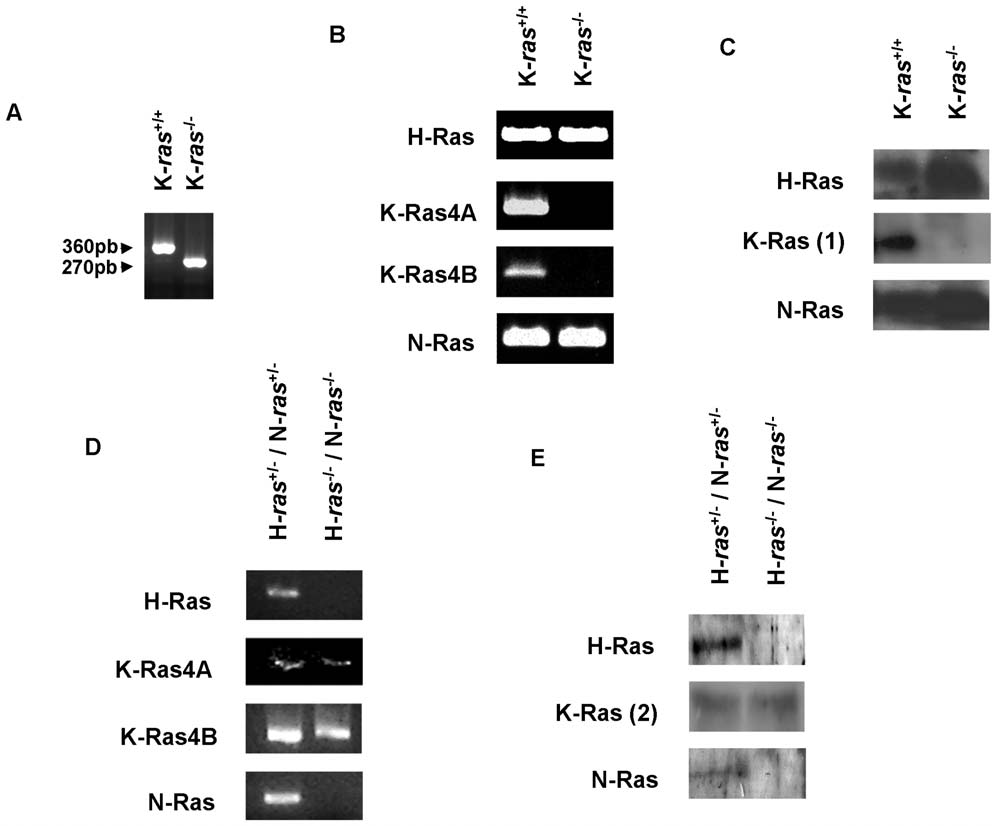
Genotypic and protein characterization of H*ras* ^−/−^,N*ras* ^−/−^ and K*ras* ^−/−^ fibroblasts by PCR and Western blot. (A) DNA PCR of K-*ras* ^−/−^ fibroblasts cultures; K-*ras* ^−/−^ fibroblasts are identified by a 270-bp product, and wild type fibroblasts by a 360-bp product (see methods section); mRNA (B) and protein (C) expression of Ras isoforms in K-*ras* ^−/−^ fibroblasts cultures; mRNA expression of H, N, K-Ras4A and K-Ras4B isoforms, evaluated by PCR (D), and protein expression (E) of H, N, and K-Ras isoforms, evaluated by Western blot, in H*ras* ^−/−^,N*ras* ^−/−^ fibroblasts. K-Ras (1): mouse anti-human K-Ras (H3845-M01) from Abnova; K-Ras (2): mouse anti-mouse K-Ras (R3400) from Sigma.

**Figure 4:**
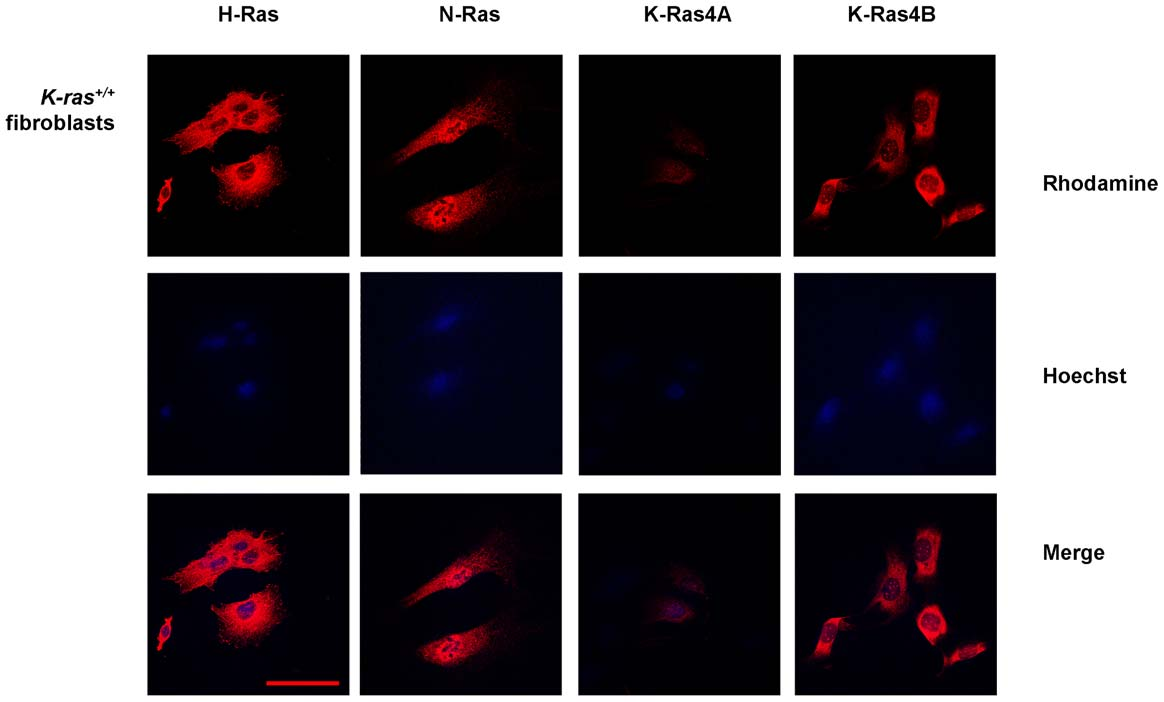
Immunofluorescence analysis of cellular distribution of Ras isoforms in K-*ras* ^+/+^ fibroblasts. Pictures show H-Ras, N-Ras, K-Ras4A and K-Ras4B expression) in K-*ras* ^+/+^ fibroblasts. The rhodamine red-labeled proteins are Ras isoforms, while the blue-labeled staining is Hoechst-stained DNA. Magnification: 630×. Scale bar: 30 µm. Antibodies: H-Ras, sc-520; K-Ras4A, sc-522; K-Ras4B, sc-521; N-Ras, sc-519.

**Figure 5:**
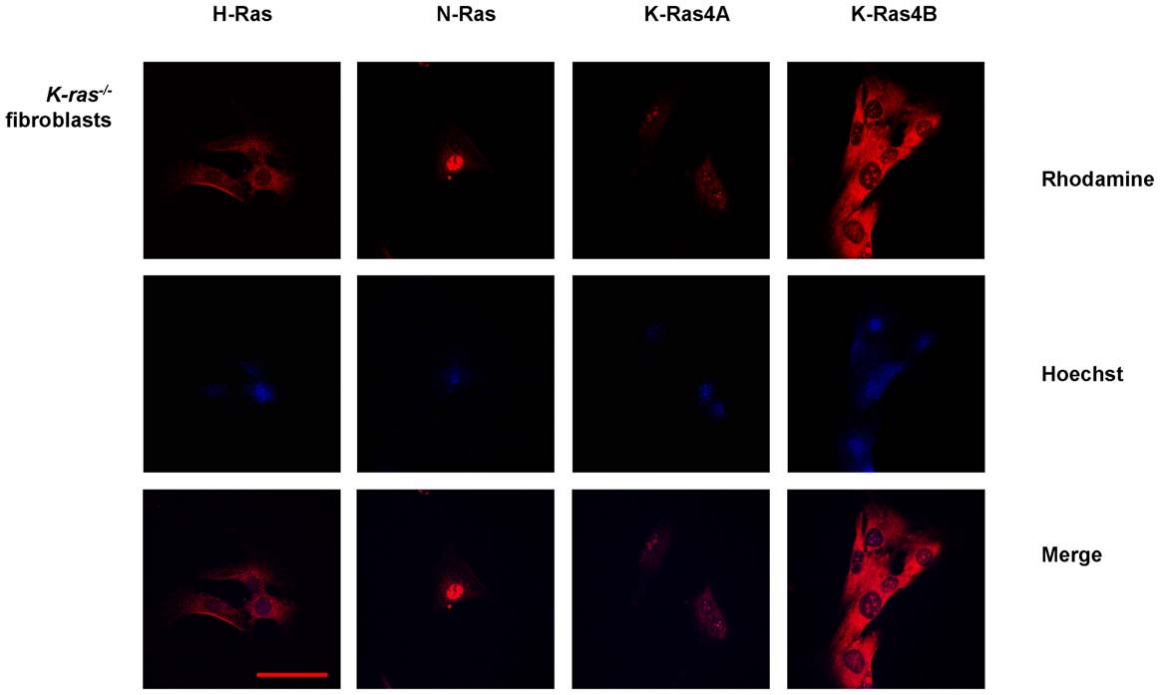
Immunofluorescence analysis of cellular distribution of Ras isoforms in K-*ras* ^−/−^ fibroblasts. Pictures show H-Ras, N-Ras, K-Ras4A and K-Ras4B expression) in K-*ras* ^−/−^ fibroblasts. The rhodamine red-labeled proteins are Ras isoforms, while the blue-labeled staining is Hoechst-stained DNA. Magnification: 630×. Scale bar: 30 µm. Antibodies: H-Ras, sc-520; K-Ras4A, sc-522; K-Ras4B, sc-521; N-Ras, sc-519.

**Figure 6:**
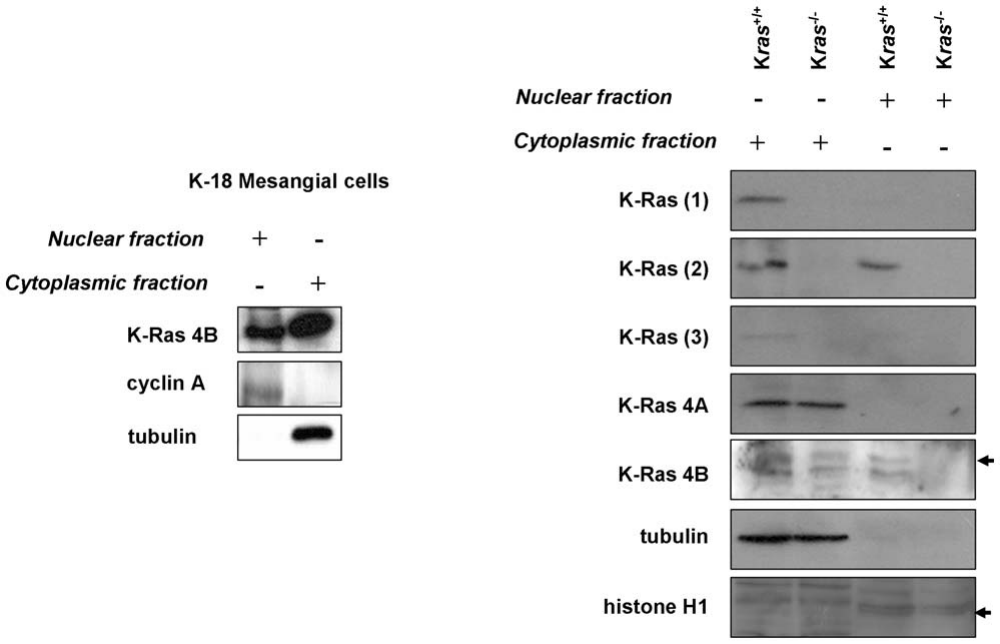
K-Ras expression analysis in nuclear and cytoplasmic fractions of human mesangial cells and K-*ras* ^−/−^ fibroblasts by western blot. K-Ras (1): mouse anti-human K-Ras (H3845-M01) from Abnova; K-Ras (2): mouse anti-mouse K-Ras (R3400) from Sigma; K-Ras (3): mouse anti-human K-Ras (sc-522) from Santa Cruz Biotechnologies. Cyclin A, histone H1 and tubulin were used as purity controls of nuclear (cyclin A, histone H1) and cytoplasmic fractions (tubulin), respectively.

The next step in our study was to reproduce the same set of experiments in K-Ras KO fibroblasts. The absence of K-Ras expression in K-*ras* ^−/−^ fibroblasts, as well as the only expression of K-Ras in H-*ras* ^−/−^/N-*ras* ^−/−^ fibroblasts was confirmed by PCR and western blot analysis. Figure 3 shows genotypic characterization and H-, N-, K-Ras4A and K-Ras4B isoforms expression in K*ras* ^−/−^ (Figure 3a–c) and H-*ras* ^−/−^/N-*ras* ^−/−^ fibroblasts (Figure 3d–e) by DNA and RNA PCR and Western blot. The immunofluorescence analysis showed the appearance of H-Ras, N-Ras and both K-Ras isoforms in the plasma membranes and K-Ras4B immunostaining in nuclei of wild type (Figure 4) and, surprisingly, in nuclei of K-*ras* ^−/−^ fibroblasts (Figure 5), with the same pattern of what was supposed to be K-Ras4B staining that we previously observed in H-*ras* ^−/−^/N-*ras* ^−/−^ fibroblasts and human mesangial cells. The sc-522 antibody against K-Ras4A also showed a fainter positive staining in both membranes and nuclei from K-*ras* ^−/−^ fibroblasts (Figure 5). Thus, our data clearly demonstrates that sc-521 and sc-522 antibodies do not specifically recognize K-Ras isoforms. K-Ras4B expression was detected by western blot in nuclei from human mesangial cells using the sc-521 antibody. Moreover, three different antibodies directed against K-Ras proteins (H3845-M01, sc-521, sc-522) detected K-Ras4B expression in nuclear extracts from K-*ras* ^−/−^ fibroblasts (Figure 6). However, other antibody directed against K-Ras (R3400, Sigma) detected its expression in nuclear fractions by western blot (Figure 6). On the other hand, the sc-521 antibody directed against K-Ras4B showed a faint signal in cytosolic and nuclear extracts from K-*ras* ^+/+^ fibroblasts but it did not show a positive signal by immunoblot in nuclear extracts from K-*ras* ^−/−^ fibroblasts (Figure 6). Moreover, the sc-522 antibody against K-Ras4A showed a positive signal in the cytosolic fraction from K-*ras* ^−/−^ fibroblasts (as we previously observed by immunostaining). Unfortunately, the antibodies which were suitable for immunofluorescence analysis did not show a good signal when used for immunobloting, and the appropriate antibodies for western blot detection were useless to perform immunofluorescence studies.

Tubulin, cyclin A and histone H1 expression were used as controls to identify the purity of cytoplasmic and nuclear preparations, respectively. However, it is almost unfeasible the complete isolation and purification of nuclear fractions, considering the continuous nature of the nuclear envelope with the endoplasmic reticulum (ER), and taking into account that 4–6% of these nuclear fractions are cytoplasmic contaminations (mainly ER) which are frequently stuck to the external nuclear membrane, as it was early described by Puddington et al. [14]. Moreover, most of the recent studies in the literature do not show controls for ER contamination in their nuclear fraction. Even the isolation of nuclear fractions by sucrose density gradient ultracentrifugation showed similar results than with the CelLytic Nuclear Extraction Kit, and traces of cytoplasmic contamination may be present (although in the nuclear fractions there was almost no expression of tubulin, which was used as purity control). Thus, it may well be that the nuclear fractions had some contamination with ER-associated proteins, attributing to the nucleus some Ras isoforms present in the ER [15].

Finally, we evaluated by ELISA whether the presumed nuclear K-Ras was able to bind Raf-RBD, Ras binding domain (active state) or not (inactive state). Whereas Ras bound to Raf-RBD is detected in cytoplasmic fractions (18.15±0.92 luminescence arbitrary units (LAU) vs. 7.38±0.74 LAU corresponding to the blank samples), our data shows that Ras is not bound to Raf-RBD in nuclear extracts from human mesangial cells as LAU were similar to those of the blank samples. In order to discard that Ras activity was not detectable in nuclear extracts because of the inactivity of this putative nuclear K-Ras, we also evaluated the presence of nuclear active Ras in the tumoral cell line HeLa, which express constitutively-active Ras. A small amount of Ras bound to Raf-RBD was detected in HeLa nuclear extracts (12.49±1.27 LAU vs. 17.32±2.42 LAU in HeLa cytoplasmic fractions), but this detection is probably due to endoplasmic reticulum contamination in the nuclear fractions. As a positive control we used an extract of EGF-treated HeLa cells, and this extract gives values over 32.78±2.21 LAU. Thus, we may suggest that if any Ras isoform is present in mesangial cells nuclei, this putative nuclear Ras is not able to bind Raf.

Although it has been suggested that the C-terminus of K-Ras4B acts as a nuclear localization signal [16], there are only two studies in the literature showing the presence of any Ras isoform in nuclei of eukaryotic cells. Immunofluorescence and electron microscopy studies identified overexpressed H-Ras and Rap-1 in or around nuclei of transformed cells [11], [17], although the functional significance of these findings is unknown. RRP22, a Ras-related protein, was found in the nucleolus [18], and recently, Birchenall-Roberts *et al.* [12], using confocal microscopy and biochemical analysis, showed K-Ras4B staining in the nucleoli of human fibroblasts suggesting a physical and functional association between K-Ras4B and nucleolin, a pleiotropic regulator of cellular processes. However, in the present study we have demonstrated that the antibodies used by Birchenall-Roberts *et al.* (sc-521 and sc-522) do not specifically recognize K-Ras proteins as they produce a clear immunostaining in K-*ras* ^−/−^ fibroblasts.

Thus, our study in K-Ras KO fibroblasts, as well as in fibroblasts that only expressed the K-Ras isoform, shows that there are no definitive evidences confirming the expression of K-Ras proteins in the nuclei of fibroblasts, because the only study published at the moment has been performed with an antibody that does not recognize specifically the K-Ras4B isoform. K-Ras in nuclei, if present, should be in its inactive form (not attached to Raf-RBD). The confirmation of the presence of nuclear forms of K-Ras is such an important finding for eukaryotic cells that makes necessary to be cautious and control exhaustively all experimental conditions.

## Materials and Methods

### Ethics statement

Mice were kept in an aseptic atmosphere with controlled temperature and light cycles in the facilities of the Animal Experimentation Service of the University of Salamanca, with free access to food and water; studies in mice were approved by the Bioethics Committee of the University of Salamanca, and animals were treated following the Recommendations from the Declaration of Helsinki and the Guiding Principles in the Care and Use of Animals stated in the international regulations and in the following European and national institutions: Conseil de l′Europe (published in the Official Daily N. L358/1–358/6, 18th December 1986), and Spanish Government (published in Boletín Oficial del Estado N. 67, pp. 8509–8512, 18th March 1988, Royal Decree Law 223/1988, from march 14 and decree from October 13 1989, and Boletín Oficial del Estado N. 256, pp. 31349–31362, 28th October 1990).

### Cell culture

We have analyzed Ras isoforms expression in different cell types: human mesangial cells and fibroblasts; we analyzed immortalized human mesangial cells (K18 line) which were obtained as described by Banas et al. [19]; K-Ras KO fibroblasts (K-*ras* ^−/−^) were cultured from K-*ras* ^−/−^ mouse embryos obtained from mating of K-*ras* ^+/−^ mice. Embryos, which were recovered at day *post coitum* 10, were genotyped by PCR as later described, mechanically minced and treated with trypsin-ethylenediaminetetraacetic acid (EDTA) 0.25% (Gibco-BRL, Cheshire, UK) for 30 min before plating. Immortalized cultures that survived crisis after 15–20 passages were identified and cloned and their genotypes reconfirmed by PCR analysis, as later described. Expression of Ras protein isoforms was monitored by immunoblotting with specific antibodies directed against H-Ras (sc-520), N-Ras (sc-519), K-Ras (sc-30), K-Ras 4A (sc-522), K-Ras 4B (sc-521) from Santa Cruz Biotechnologies (Santa Cruz, CA, USA), K-Ras (H3845-M01) from Abnova (Heidelberg, Germany) and K-Ras (R3400) from Sigma (Madrid, Spain). At least two sets of independently generated, immortalized fibroblast cell lines (originated from different embryos) were used for analysis of the K-*ras* ^−/−^ genotype. H-*ras* ^−/−^/N-*ras* ^−/−^ embrionary fibroblasts were obtained from mice genetically deficient for H- and N-Ras isoforms (H-*ras* ^+/−^/N-*ras* ^+/−^ and H-*ras* ^−/−^/N-*ras* ^−/−^) as previously described [7]. Embrionary fibroblasts and human epithelial cervical cancer (HeLa) cells were grown in DMEM medium (Bio Whittaker Labs, Rockland ME, USA) supplemented with 10% fetal calf serum (FCS, Bio Whittaker Labs), 0.66 µg/ml penicillin and 60 µg/ml streptomycin sulfate (Bio Whittaker Labs), in an atmosphere of 95% air/5%CO2 at 37°C. Mesangial cells were grown in RPMI 1640 (Bio Whittaker Labs) supplemented with 10% FCS, 1 mM L glutamine, 0.66 µg/ml penicillin, 60 µg/ml streptomycin sulfate, 5 µg/ml insulin, 5 µg/ml transferrin and 5 ng/ml selenium, in an atmosphere of 95% air/5% CO2. Cells were seeded in 100 mm Petri dishes (Nunc, Roskilde, Denmark) for Western blot and Elisa assays, and in cover slips (Nunc) for immunofluorescence studies, respectively.

### Polymerase Chain Reaction (PCR)

Fibroblast genotyping was performed after DNA extraction from cell cultures with DNA lysis buffer (50 mM Tris pH 7.5, 5mM EDTA pH 8.0, 100 mM NaCl, 1 mM dithiothreitol, 0.5 mM spermidin, and 10 mg/ml proteinase K). PCR was carried out using the following primers: K-*ras* IMR393 (common K-*ras* primer), 5′- AGG GTA GGT GTT GGG ATA GC - 3′, K-*ras* IMR394 (wild type K-*ras* primer), 5′- CTC AGT CAT TTT CAG CAG GC - 3′, and K-*ras* IMR395 (mutant K-*ras* primer), 5′- ACG AGA CTA GTG AGA CGT GC - 3′. The primer set of K-*ras* IMR393 and K-*ras* IMR395 amplify a 270-bp product in K-*ras* ^−/−^ fibroblasts, and the combination of K-*ras* IMR393 and K-*ras* IMR394 amplify a 360-bp product in wild type fibroblasts. Oligonucleotides were used in a 25-µl reaction mixture with 1 µl of DNA and 2 units of Taq polymerase. In these reactions, DNA was denatured for 3 min at 94°C and amplified for 35 cycles at 94°C for 30 s, 62.5°C for 30 s and 72°C for 30s, followed by an elongation cycle of 72°C for 2 min, using a BioRad Thermal Cycler. Amplified products were analyzed by electrophoresis in 1.5% agarose gels. The genotypes were subsequently confirmed by rt-PCR with primers against K-*ras*4A and K-*ras*4B, and by western blotting.

Total RNA was extracted and purified from cell cultures using Tri-reagent (Molecular Research, Cincinnati, OH, USA). First-strand cDNA was generated using M-MLV Reverse Transcriptase and oligo(dT) as described by the manufacturer. RT-PCR was performed using the next primers: for H-*ras*, 5′-AAG CTT GTG GTG GTG GGC GCT AAA GGC- 3′ and 5′- CTT TCA CCC GCT TGA TCT GCT CCC TGT ACT - 3′, corresponding to positions 13 to 39 and 284 to 313 of the coding sequence; for N-*ras*, 5′ - CCA GGA TTC TTA CCG AAA GCA AGT GGT G-3′ and 5′ - CCT GTA GAG GTT AAT ATC TGC AAA TG-3′, corresponding to positions 4 to 31 and 162 to 187 on exon II; and for K-*ras*, 5′- AGT ACG ACC CTA CGA TAG AGG ACT CCT-3′, bp 92 to 118, 5′ - CAA TCT GTA CTG TCG GAT CTC TCT CAC C - 3′, specific for K-*ras*4A bp 477 to 504, and 5′- CTA ATG TAT AGA AGG CAT CGT CAA CAC CC - 3′, specific for K-*ras*4B, bp 450 to 478, of their respective coding sequences. For GAPDH, 5′- TGA AGG TCG GTG TGA ACG GAT TTG GC - 3′, and 5′- CAT GTA GGC CAT GAG GTC CAC CAC - 3′. Cycling conditions for *ras* isoforms were 95°C for 4 min followed by 30 cycles of 94°C for 1 min, 62°C for 1 min, and 72°C for 1 min, followed by an elongation cycle of 72°C for 10 min; and for GAPDH were 94°C for 5 min followed by 28 cycles of 94°C for 1 min, 60°C for 1 min, and 72°C for 1.5 min, followed by an elongation cycle of 72°C for 5 min.

### Immunofluorescence analysis

Cells in cover slips were fixed with 4% paraformaldehyde (Sigma, St Louis MO, USA), washed with phosphate buffered saline with calcium and magnesium (PBS Ca-Mg: 1mM CaCl_2_, 1 mM MgSO_4_, 0.81% ClNa, 2.6 mM PO_4_H_2_K, 4.1 mM PO_4_HNa_2_), permeabilyzed with 0.1% Triton X-100, 0.2% BSA and 0.5% sodium azide, quenched with NH_4_Cl 50 mM in PBS Ca-Mg, blocked with 10% normal goat serum (NGS, Santa Cruz Biotechnology, Santa Cruz CA, USA) in PBS Ca-Mg for 30 min, and incubated during 2 hours with primary antibodies (dilution 1/50–1/100) in PBS Ca-Mg with 2% NGS: rabbit anti-human H-Ras (sc-520), rabbit anti-human K-Ras2A (sc-522) and 2B (sc-521) (here referred as 4A and 4B), and rabbit anti-human N-Ras (sc-519). According to the distributor, the antibodies used in this study were supposed to be specific for the corresponding proteins and guaranteed not to cross-react with each other. Blocking peptides were also used to check the specificity of every antibody (data not shown). Later, cells were incubated 30 min with goat anti-rabbit Cy3 (Jackson Immunoresearch, West Grove PA, USA) (dilution 1/1000) in PBS Ca-Mg with 2% NGS or normal donkey serum (Sigma) in a dark chamber. Nuclei staining was performed by 5 min incubation with 2µM Hoechst 33258 (Molecular Probes) in a dark chamber. Cover slips were mounted on slides using Prolong gold antifade (Molecular Probes). Confocal images were made using a Zeiss Axiovert 200M microscope and a Zeiss LSM 510 confocal module, with a HeNe laser with 543-excitation for rhodamine and Hg laser with 365-excitation for DAPI. All images were obtained with identical parameters for intensity, pinhole aperture, etc.

### Western blot

Nuclei extracts were isolated by sucrose gradient ultracentrifugation. Cells were washed twice with cold PBS, scrapped with buffer A (250 mM sucrose, 20 mM Hepes pH 7.4, 1 mM EDTA and protease inhibitors: 0.1 mM PMSF, 2µg/ml leupeptin, 2 µg/ml pepstatin) and disrupted by 20 passages through a 23 gauge needle; then cells were centrifuged at 500 g for 5 min, and the pellet was washed with buffer I (10 mM Hepes-NaOH pH 7.9, 10 mM KCl, 1.5 mM MgCl_2_, 0.5 mM dithiothreitol plus protease inhibitors); cells were again centrifuged at 500 g for 5 min and the nuclei pellet was washed with buffer I with 0.25 M sucrose and protease inhibitors. After another centrifugation, nuclei were resuspended in buffer I and centrifuged at 100000 g for 1 h over a 1.8 M sucrose cushion prepared in buffer I. The nuclei pellet was washed in buffer I without sucrose by spinning 5 min at 1000 g, and resuspended in buffer I, 0.1% (v/v) Nonidet P-40 and incubated at 4°C with intermittent mixing for 10 min. Nuclei was harvested twice by 5 min spin at 500 g and washed in buffer I; finally, nuclei was collected in nuclei storage buffer (10 mM piperazine-N,N′-bis(2-ethanesulfonic acid) pH 7.4, 5 mM EGTA, 80 mM KCl, 20 mM NaCl, 50% glycerol, 250 mM sucrose, 1 mM dithiothreitol, 0.2 mM spermine, 0.5 mM spermidine) and stored under liquid nitrogen.

Nuclei extracts were also obtained with the CelLytic Nuclear Extraction Kit (Sigma): cells swell with hypotonic buffer (10 mM HEPES, pH 7.9, 1.5 mM MgCl_2_ and 10 mM KCl) supplemented with 1 mM dithiothreitol (DTT) and protease inhibitor cocktail, then cells are disrupted with 0,6% Igepal, the cytoplasmic fraction is removed, and the nuclear proteins are released from the nuclei by a high salt extraction buffer (20 mM HEPES, pH 7.9, 1,5 mM MgCl_2_, 0.42 M NaCl, 0,2 mM EDTA, and 25% glycerol) supplemented with 1 mM dithiothreitol (DTT) and protease inhibitor cocktail [20].

Protein content of nuclei and cytoplasmic extracts was determined by the Lowry assay (Dc protein assay, BioRad, Hercules CA, USA). Electrophoresis and blotting was performed as previously described [21] with the following primary antibodies: mouse anti-human K-Ras (H3845-M01, Abnova, and sc-30, Santa Cruz Biotechnologies), mouse anti-mouse K-Ras (R3400, Sigma), rabbit anti-human K-Ras4A (sc-522), rabbit anti-human K-Ras4B (sc-521), rabbit anti-human H-Ras (sc-520), rabbit anti-human N-Ras (sc-519) (dilution: 1/200), rabbit anti-human cyclin A (sc-571, Santa Cruz Biotechnologies), goat anti-human histone H1 (sc-8615, Santa Cruz Biotechnologies) (dilution: 1/1000) and mouse anti-mouse tubulin (OP06) (dilution 1/1000) from Calbiochem (San Diego CA, USA). After incubation with the corresponding secondary antibody (goat anti-rabbit and goat anti-mouse IgG (H+L) horseradish peroxidase (HRP) conjugated antibodies from BioRad and donkey anti-goat from Santa Cruz Biotechnologies) at dilutions 1/2000–1/10000, membranes were finally incubated with a chemiluminescent reagent (ECL detection reagent, Amersham Biosciences, Piscataway NJ, USA) and developed signals were recorded on x-ray film (Hyperfilm, Amersham Biosciences, Piscataway NJ, USA).

### ELISA

We evaluated the presence of Ras bound to Raf-RBD (active Ras) in nuclei of human mesangial cells and human tumoral HeLa cells, using an ELISA method (Ras GTPase Chemi ELISA, Active Motif, Rixensart, Belgium); this kit only detected Ras activation in human cells. 50 µg of 4 different nuclear and cytoplasmic extracts were analyzed. 25 µg HeLa whole cell extract (EGF treated) was used as positive control. Chemiluminescence was read using a luminometer (Thermo Luminoscan Ascent, Waltham MA, USA) within 15 minutes to minimize changes in signal intensity.
